# A Challenging Diagnosis of Systemic Lupus Erythematosus with Status Epilepticus

**DOI:** 10.7759/cureus.4783

**Published:** 2019-05-30

**Authors:** Pulwasha M Iftikhar, Maham Munawar, Choudhary A Hasan, Mohammed FaisalUddin, Aly Cohen

**Affiliations:** 1 Health Sciences, St. John's University, New York, USA; 2 Internal Medicine, Dow University of Health Sciences (DUHS), Karachi, PAK; 3 Internal Medicine, Deccan College of Medical Sciences, Hyderabad, IND; 4 Internal Medicine, CentraState Medical Center, Freehold, USA

**Keywords:** seizure, status epilepticus, neuropsychiatric manifestation, autoimmune, systemic lupus erythematosus (sle)

## Abstract

Systemic lupus erythematosus (SLE) is a chronic autoimmune disorder of unknown pathogenesis. In SLE, the body’s immune system mistakenly attacks healthy tissues and organs thereby involving multiple body systems including joints, skin, blood, brain, heart, and lungs. SLE has a wide variation in the symptoms, hence making the diagnosis more challenging at the time of initial presentation. Sometimes, the patient presents with Status epilepticus (SE) without prior history of epilepsy, as SE is common at the beginning in the course of SLE. In this report, there is a case showing correlation of seizures with SLE, without prior history of epilepsy. A 43-year-old female presented in the emergency department of the hospital with SE. Her previous medical and family history for epilepsy was unremarkable. The patient had high titers for positive anti-nuclear antibody (ANA), while other autoimmune workup was negative. A complete evaluation of the symptoms and investigations revealed that she met the criteria of American College of Rheumatology (ACR) for the diagnosis of SLE. Hence, physicians should be diligent with regards to the variations in the initial presentation and complications of SLE. With the advancement in treatment modalities of SLE, it can be managed successfully, if diagnosed early.

## Introduction

Systemic lupus erythematosus (SLE) is a chronic autoimmune disorder of unknown pathogenesis. In SLE, the body’s immune system mistakenly attacks healthy tissues and organs thereby involving multiple body systems including joints, skin, blood, brain, heart, and lungs [1]. The exact cause of the disease is unknown but autoantibodies are considered as the focal culprit of the disease, which can be detected years before the clinical manifestation [2]. The symptoms are often vague and gradual in onset, hence delaying the diagnosis and making it more challenging. SLE is diagnosed, based on both clinical and laboratory features. The American College of Rheumatology (ACR) has developed a classification criterion for Lupus and it is used as an aid in the diagnosis of this complex multi-organ system disease [3]. The initial presentation of SLE varies widely secondary to the involvement of different organ systems.

SLE affects the nervous system at multiple levels, thus causing variable behavioral, psychiatric and neurological manifestations. Neuropsychiatric symptoms present in 10-80% patients prior to SLE diagnosis or during course of the disease [4]. These symptoms include headache, mood disorders, cognitive decline and seizures. Exact mechanisms of nervous tissue damage have not been discovered, but almost half of the seizures in SLE are associated with metabolic disorders and infections. Cytokines IL1, IL6 and TNF alpha activate the hypothalamic pituitary axis (HPA), and subsequently decrease the seizure threshold [4,5]. In SLE, seizures are usually generalized tonic-clonic. It is the most inauspicious feature of the disease, hence leading to bad prognosis [5,6]. We herein report a case of a 43-year-old female with SLE who presented with Status epilepticus (SE) for the first time without history of epilepsy.

## Case presentation

A 43-year-old female presented in the emergency department of the hospital with SE. Her previous medical and family history for epilepsy was unremarkable. However, she had a six-month history of migraines for which she was treated for by a neurologist. On clinical examination, she had tonic contractions and clonic jerks, her pulse was 55 beats per minute, Glasgow coma scale (GCS) was 4/15, blood pressure was 80/50 (supine), and she had brittle nails, alopecia, and mouth ulcers.

The initial laboratory investigation revealed white blood cell (WBC) count of 4.7 x 10^3^/mm^3^, a platelet count of 241 x 10^3^/mm^3^ and a hemoglobin level of 8.6g/dl. The serum electrolytes, metabolic screening of urine and blood, lactic acid, pyruvic acid were normal. Renal function tests and liver function tests were found to be in the normal range as well. A lumbar puncture performed revealed no WBCs or protein and glucose was within the normal range. Serological studies revealed positive anti-nuclear antibody (ANA). Whereas, anti-double stranded DNA (anti-dsDNA), antiphospholipid antibodies, anti N-methyl-D-aspartate (NMDA) were negative. There was no evidence of viral, bacterial, fungal infection found in the results from the cerebrospinal fluid (CSF) and blood. Toxicological studies were also negative. Brain MRI and brain magnetic resonance angiography (MRA) did not reveal any underlying pathology. Also, an electroencephalogram was performed, which showed generalized epileptic activity.

Conventional antiepileptic drugs failed to control the seizures and therefore intravenous midazolam infusion was started, which controlled the seizures successfully. The patient was intubated in order to prevent respiratory failure as her GCS was 4/15. She was further managed with IV fluids and a nasogastric tube was attached to maintain parenteral nutrition. There was no improvement in the GCS for six days. Therefore, she was given a continuous infusion of midazolam to control her subclinical seizures and she was monitored through an electroencephalogram. Methylprednisolone IV 1 gm was infused as autoimmune encephalitis was not initially ruled out. GCS of the patient improved to 15/15 on sixth day. As subclinical seizures subsided, respiratory support was removed and midazolam was discontinued. She was then discharged on antiepileptic drugs and advised to continue anti-migraine medications.

Shortly after discharge, she had episodes of muscle twitching, so her antiepileptic drugs were adjusted accordingly. She also developed a malar rash for which a rheumatologist was consulted. He established the diagnosis of SLE on the basis of refractory cytopenia, positive ANA, arthritis, mouth ulcers, malar rash, seizures, and migraine. She was treated with IV methylprednisolone 1 gm for five days. Rituximab was administered in the dosage of 2 gm (1 gm each one week apart), which improved her condition drastically. She was followed with oral prednisolone 45 mg, which was tapered gradually. The patient's cytopenia, arthritis, rash and migraine improved after treatment. The patient is now overall stable and the lupus flares secondary to any sort of infections are successfully managed with corticosteroids.

## Discussion

SLE is a chronic autoimmune disorder that often involves multiple systems, hence described as a ‘disease with a thousand faces' [5]. The diverseness of the clinical manifestations continually makes the diagnosis complex [1]. Prevalence and mortality of lupus are far more common in people belonging to South East Asian ethnicity than Caucasians, and the disease is predominant amongst the female gender [6-8].

The ACR has categorized the neuropsychiatric symptoms of SLE as a cerebrovascular disease with cognitive dysfunction, seizures, psychosis, and peripheral nervous system disorder [9]. All the symptoms show extensive recurrence rate. Studies conducted previously prove that the occurrence of epilepsy is three times more common in people with SLE [10-12]. Park et al. reported a case of 17-year-old girl who presented with status epilepticus at onset of SLE [13]. Similarly, in this case the patient presented with SE without a positive history of epilepsy and SLE. The exact pathogenesis of the disease is unknown but is often correlated to inflammatory cytokines, auto antibodies, specific and nonspecific complexes that accelerates the neuronal damage. Cardinal central nervous system involvement serves as a diagnostic challenge mainly because the pattern and symptoms of lupus involving central nervous system could be focal, diffuse or a combination of both [11]. This case emphasizes the neurological manifestations associated with SLE and the need to perform a thorough clinical, radiological and laboratory workup at the time of grand mal epileptic seizures to prevent its recurrence and progression in certain autoimmune disorders like lupus that ordinarily involves the central nervous system.

SLE could present with other autoimmune disorder as well, which could possibly complicate the treatment. The study conducted by Sordia-Ramírez et al. showed the presence of neuropsychiatric symptoms in a patient with Dyke-Davidoff-Masson syndrome [14]. The seizures in this patient presented due to cortical hypotrophy in the left medial temporal lobe. However, in our patient, no pathological finding was discovered as a cause of SE nor was there a presence of positive history of epilepsy. Therefore, a patient presenting with SE must undergo complete clinical and laboratory investigation to exclude any systemic autoimmune disease. The diagnosis of SLE could be difficult because it is established through clinical findings which not only show a wide variation but commonly exhibit at a later stage of the disease. In the previous years, vast treatment options have been introduced for SLE which could alleviate the symptoms thereby decreasing the mortality rate [15].

## Conclusions

SLE is a disease affecting nearly every organ system making its diagnosis a troublesome task even for the skilled physicians. At times, the patient presents with SE without prior history of epilepsy, as SE is common at the beginning in the course of SLE, hence physicians must be incredibly knowledgeable regarding the complications of SLE and physicians should not confuse it with neuropsychiatric disorders. They must carry out a detailed workup to rule out SLE before it presents with other obvious symptoms.
